# Ochratoxin A and Sterigmatocystin in Long-Ripened Grana Cheese: Occurrence, Wheel Rind Contamination and Effectiveness of Cleaning Techniques on Grated Products

**DOI:** 10.3390/toxins14050306

**Published:** 2022-04-27

**Authors:** Amedeo Pietri, Giulia Leni, Annalisa Mulazzi, Terenzio Bertuzzi

**Affiliations:** Department of Animal Science, Food and Nutrition, Università Cattolica del Sacro Cuore, 29122 Piacenza, Italy; amedeo.pietri@unicatt.it (A.P.); giulia.leni@unicatt.it (G.L.); annalisa.mulazzi@unicatt.it (A.M.)

**Keywords:** ochratoxin A, sterigmatocystin, grated cheese, long-ripened grana cheese

## Abstract

A survey on the occurrence of ochratoxin A (OTA) and sterigmatocystin (STC) in grated cheese products obtained from hard grana-type cheeses was carried out, where 107 grated products were collected in retail outlets and analysed. OTA and STC were found in 48.6% and 94.4% of the samples, in a range from <LOD to 25.05 µg kg^−1^ and from <LOD to 6.87 µg kg^−1^, respectively. STC was detected in all the OTA-contaminated samples. The OTA and STC occurrence in cheese is due to environmental contamination during ripening, leading to fungal growth and mycotoxin production on the cheese surface. This statement was confirmed by analysing the surface of 16 hard grana cheese rinds, which resulted contaminated by both OTA and STC, with concentration ranging from 3 to 370 µg kg^−1^. This finding demonstrates that rind inclusion increases the mycotoxin concentration in grated cheeses. The mycotoxin level significantly decreased from the surface (0–1.5 mm) to inner parts of cheese rinds (1.5–4.5 mm). Industrial wheel-cleaning techniques can represent a useful treatment to reduce both toxins in grated cheese products.

## 1. Introduction

Mould growth commonly occurs during the production and ripening of cheeses. Sometimes, mould occurrence is necessary for providing a unique flavour to the final cheese, as in the case of non-toxigenic *Penicillium roqueforti* strains, used for the production of blue mould cheeses. However, the development of certain filamentous fungi on the cheese surface can also negatively affect their quality, causing discoloration, poor appearance and off-flavours [1]. As a matter of fact, fungi belonging to several genera *(Penicillium*, *Aspergillus*, *Cladosporium*, *Geotrichum*, *Mucor*, *Trichoderma Acremonium*, *Alternaria*, *Aureobasidium*, *Botrytis*, *Epicoccum*, *Eurotium*, *Exophiala*, *Fusarium*, *Gliocladium*, *Lecanicillium*, *Rhizopus and Wallemia)* have already been identified as being responsible for cheese spoilage [2]. Among these genera, several species are able to not only optimally grow in environmental conditions similar to those used for cheese ripening (15–25 °C) [3], but also to produce toxic secondary metabolites known as mycotoxins [4]; therefore, mould growth on cheese surface could be a concrete risk for consumers’ health.

Cheese has been reported to contain mycotoxins, such as aflatoxin M_1_, ochratoxin A (OTA), sterigmatocystin (STC) and citrinin [5]. OTA is produced by various fungi of the genera *Aspergillus* and *Penicillium* and presents nephrotoxic, teratogenic, immunogenic, hepatotoxic and carcinogenic activities [6]. OTA is classified as a Group 2B carcinogen by the International Agency for Research on Cancer (IARC) since 1993, indicating it as a possible human carcinogen [7]. Furthermore, in the last decades, published data have also demonstrated the genotoxicity of OTA, its role in oxidative stress and have identified epigenetic factors involved in OTA carcinogenesis [8,9], thus suggesting an upgrade of its IARC classification [10]. STC is considered an emerging mycotoxin and is produced by fungi of the genera *Aspergillus, Bipolaris*, *Chaetomium* and *Emiricella* [11]. Several studies have recognised STC as a potential carcinogen, mutagen and teratogen toxin, and the IARC classified STC as Group 2B [12] since 1987.

In recent years across Europe, the production and consumption of grated cheeses, which are usually obtained from long-ripened cheeses, has been continuously growing [13]. In particular, about 25% of total hard cheese production in Italy is currently destined to grated cheeses [13]. The whole wheel of cheese is grated after surface cleaning; for grana-type cheeses, rind cannot exceed 18% (*w*/*w*) of the grated product. Grated cheeses have already been reported as food products with a potential mycotoxin contamination. Biancardi et al., in 2013 [14], detected the presence of OTA in grated cheeses collected in retail outlets located in Northern Italy. This first survey, performed on 40 grated cheese products, showed that 15% of the samples were contaminated by OTA. More recently, Altafini et al. [15] showed a similar rate of OTA contamination in grated cheeses collected in the urban area of Bologna (Italy). Unfortunately, to our knowledge, no data on the occurrence of STC in grated cheese products is available so far, even if its presence has already been reported in ripened cheeses [16].

STC and OTA occurrence in cheeses is related to environmental conditions of warehouses during ripening, rather than to endogenous contamination of raw milk. In addition, their presence in grated cheeses has already been linked to the occurrence of toxigenic fungi on the surface of cheese wheels [14,17]. These studies reported that careful monitoring of hygienic and microbiological conditions, as well as correct cleaning practices, could represent a feasible way to minimise mycotoxin contamination of cheese wheels. 

In the present work, a survey on the occurrence of OTA and STC in 107 samples of grated cheese products obtained from hard grana-type cheeses was carried out. Furthermore, migration of these mycotoxins from the surface to inner parts of hard cheese rinds was studied. Lastly, the effectiveness of industrial cleaning procedures of cheese wheels in reducing the contamination of grated hard cheeses was evaluated.

## 2. Results

### 2.1. Performances of the Methods

Recovery percentages were assessed at two spike levels (3 replicates), and the recoveries ranged between 88.1% and 94.3% for OTA and 86.7% and 92.4% for STC. Mean values of 91.6% ± 2.5% and 88.9% ± 2.3% were obtained, respectively, completely fulfilling the performance criteria fixed by Regulation (EC) 401/2006 of the Commission of the European Communities [18]. STC assay interference was evaluated by comparing the response of [^13^C_18_]-STC in sample extracts and in a standard solution, and interference ranged between 10% and 18%. As far as OTA is concerned, no interfering peaks were observed in the spiked samples and no significant peaks were found at the specific retention time in the blank samples, showing a satisfactory specificity. The LOD and LOQ were 0.05 and 0.15 µg kg^−^^1^ for both OTA and STC. Finally, the linearity of calibration curves was very satisfactory, providing coefficient of determination values (R^2^) always above 0.997.

### 2.2. Occurrence of OTA and STC in Grated Cheese Samples

A total of 107 grated cheese samples were collected at the retail level in Northern Italy over the period 2018–2020 and analysed for the presence of OTA and STC. Descriptive statistics (incidence, mean, median and range of contamination) of the results are reported in Table 1. 

Collected data show a widespread and not negligible contamination for both OTA and STC. In particular, regarding OTA, the incidence in grated cheese was 48.6% and the contamination level was very variable. Regarding STC, the mycotoxin was detected in almost all of the samples (incidence 94.4%); however, the contamination level was lower and more uniform than that of OTA. Regarding OTA, 21.5% and 10.3% of the samples showed a concentration higher than 2 and 5 µg kg^−^^1^, respectively. In 52 samples, the co-occurrence of both toxins was detected; however, interestingly, no correlation was found (r = 0.013) between the levels of OTA and STC in these samples.

### 2.3. Migration of OTA and STC from Surface to Inner Parts of the Rind

In order to investigate migration of the target mycotoxins from the surface to inner layers of hard cheese, OTA and STC were analysed in four separated layers of the rind. For each piece of rind, average contamination levels of OTA and STC on the surface and in the layers beneath are reported in Table 2. 

**Figure 1:**
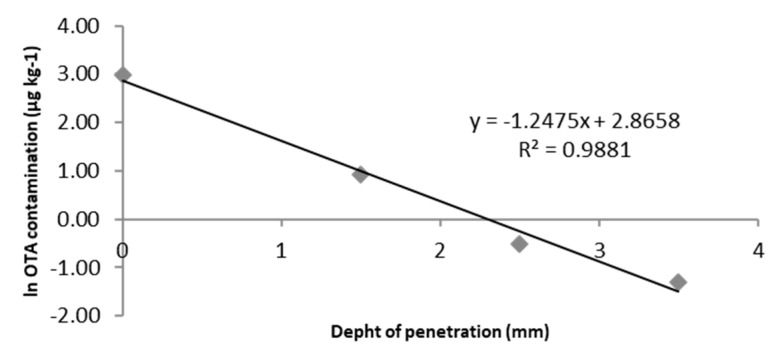
Linear correlation between depth of penetration (mm) and OTA concentration (logarithmic µg kg^−1^ transformed data) in a ripened hard cheese wheel.

Results demonstrated that OTA contamination of the surface (0–1.5 mm) was different among the wheels, ranging from 10.8 to 370.4 µg kg^−^^1^. In 25% of the samples, the OTA level exceeded 200 µg kg^−^^1^; in these wheels, the level was also high in the second layer (1.5–2.5 mm). Interestingly, a negative correlation (R = −0.697) was found between the concentration of OTA on the surface and its percentage reduction in the second layer (79% ± 10%), showing that the higher the OTA concentration in the first layer, the lower its percentage reduction in the second layer. Moreover, the concentration decreased remarkably in the third and fourth layer at all levels of OTA contamination. Considering that OTA contamination of the inner parts of cheese could be favoured by penetration of toxigenic fungi and/or possibly by the polarity of the toxin (cheese rind surface is fattier than inner layers), we evaluated if the capacity of penetration, equivalent to −∆[OTA]/∆S (with ∆S = mm of depth from the surface), was linearly correlated with the mycotoxin concentration on the surface. Based on this hypothesis, −OTA]/∆S = k[OTA]_surface_ can be expressed as −∆[OTA]/[OTA] = k∆S. Considering infinitesimal variations, the equation can be expressed as −∫(1/[OTA])d[OTA] = k∫dS. By solving this, the hypothesis can be expressed as: ln[OTA] = −kS + ln[OTA]_surface_. Plotting the ln[OTA] in function of the depth of penetration (mm), a linear correlation can be obtained. As suggested, a linear correlation (calculated for samples where OTA was detected at least up to the third layer) was obtained for each wheel sample (n = 14), with R^2^ values ranging from 0.8458 to 0.9985, and 11 wheels showed R^2^ values higher than 0.94. As an example, Figure 1 shows the linear correlation calculated in a representative cheese wheel. 

Moreover, the k value obtained for each wheel varied from 1.2475 to 1.7822, with a mean value of 1.4697 ± 0.1638. Therefore, knowing the OTA concentration on the surface and applying this equation, it is possible to approximately evaluate the OTA concentration in the layers beneath.

Regarding STC, the concentration on the surface was always much lower than OTA, ranging from 2.6 to 24.4 µg kg^−^^1^. As for grated cheese samples, no correlation with OTA was observed. The decrease of concentration in the layers beneath was significantly slower if compared to that of OTA. In fact, the average reduction percentage of STC contamination from the first layer to the second one resulted as 48% ± 16%. Furthermore, the same penetration model developed for OTA was tested for STC. R^2^ values (calculated for 11 samples) ranged from 0.8558 to 0.9913, and 8 samples presented R^2^ values exceeding 0.93. The k value varied from 0.3742 to 1.0562 mm^−^^1^ (mean value of 0.6512 ± 0.2511 mm^−^^1^), showing a higher variability if compared to OTA.

### 2.4. Efficacy of Wheel Cleaning Techniques Used in Grated Hard Cheese Production

The results regarding the wet cleaning process on OTA and STC levels are reported in Table 3. 

The washing water collected before and after the cleaning of the five wheels chosen for the trial presented a similar level of OTA and STC contamination: 0.44 and 0.39 µg kg^−^^1^ for OTA, and <LOD for STC. Warm water used in the washing plant was discarded every day, however the plant had been running for five hours at the start of the trial. Therefore, a significant OTA level was detected at T0, due to the high number of wheels processed before, and this level was not modified by the five wheels of the trial. Low concentrations of OTA and STC were found in grated cheese samples, with a mean value of 0.28 ± 0.30 and 0.17 ± 0.18 µg/kg^−^^1^ for OTA and STC, respectively. No remarkable difference was observed between these data and those obtained from commercial wheels previously processed in the plant. These results show that the selected wheels were probably less contaminated than expected.

Regarding the dry-cleaning technique, OTA and STC levels were evaluated not only on grated cheese after processing, but also on the rind powders removed during the cleaning process by scraping (applied separately both on the flat and on the round side) and brushing, and the results are reported in Table 4.

In the scraping process, OTA and STC concentrations in powders removed from the flat side resulted significantly higher (*p* < 0.01) compared to the round side of the wheel. Lower levels of OTA and STC were found in rind samples removed by brushing. These data show that a deeper scraping of the flat side of the wheel (representing about 45% of the wheel surface) should allow a significant decrease of the contamination level of grated cheeses. Furthermore, grated cheese samples obtained from the five processed wheels were analysed for OTA and STC, and the results are reported in Table 5.

As for wet cleaning, the results for OTA and STC in grated cheeses produced after dry cleaning showed a low contamination level, with a mean value of 0.14 ± 0.19 and 0.16 ± 0.12 µg kg^−^^1^ for OTA and STC, respectively. These data were similar to those of the grated cheeses obtained after wet cleaning, demonstrating that the techniques are comparable in case of low contamination levels.

## 3. Discussion

The growth of toxigenic fungi on cheese wheels represents a risk for consumers, due to the potential production of different mycotoxins. Nowadays, cheese and dairy products are only regulated for aflatoxin M_1_ [19], but other mycotoxins can contaminate these foodstuffs. The survey carried out in this work showed, as a matter of fact, detectable levels of OTA and STC in grated grana-type cheese available at the retail level. No specific regulations have been adopted for STC in food worldwide. The EU fixed specific limits for OTA in some foodstuffs (dairy products excluded), and considering the OTA limits set for cereals, dried vine fruits and spices (3, 10 and 15 µg kg^−1^, respectively), the percentages of grated cheese samples exceeding these values were 20%, 5% and 3%, respectively. OTA occurrence in grated cheeses has been recently reported by Altafini et al. [15] in 7 out of 51 samples of grated hard cheese, with concentrations ranging between <LOD and 22.4 µg kg^−1^. A similar incidence was previously reported by Biancardi et al. [14], where OTA was detected in 15% of grated cheese samples collected from retail shops in Northern Italy, with a contamination level ranging between <LOD and 54.07 μg kg^−1^. The incidence reported in these surveys is lower if compared to the one found in our survey, probably because of the higher LOQ value (1 µg kg^−1^) of the methods used in both papers.

Considering that the transfer of OTA from feed to ruminant milk is negligible, due to the hydrolytic activity of rumen bacteria, OTA occurrence in cheese is almost certainly due to environmental contamination, caused by fungal growth on the cheese surface [1,2]. This was confirmed by analysing the surface of 16 hard cheese rinds, which all resulted contaminated by both OTA and STC, with concentrations ranging between 11 and 370 µg kg^−1^ for OTA and between 3 and 26 µg kg^−1^ for STC. No contamination was found in the inner part of the same wheels. Moreover, the study on the penetration capacity of these mycotoxins in the rind of grana-type cheese demonstrates that OTA and STC levels markedly decreased from the surface (0–1.5 mm) to the inner parts (1.5–4.5 mm) of the rind. These results were in agreement with Anelli et al. [20], who showed that OTA contamination significantly decreases from the rind to the core of traditional cave cheeses. The possible penetration of OTA from the rind up to the depth of 1.6 cm was also reported for French semi-hard Comte cheese, after an artificial inoculation with ocratoxigenic strains [21]. In another work, Pattono et al. [22] detected OTA on both the surface and inner part of hand-made semi-hard cheeses.

Our results clearly demonstrate that the inclusion of contaminated rinds in grated cheese products can contribute to the increase of OTA and STC levels in the final product. However, since STC and OTA occurrence in cheeses is related to environmental conditions of cheese ripening warehouses, it is of great importance to control toxigenic mould development during cheese storage. In order to reduce mould contamination of cheese, it is necessary to systematically take preventative measures aimed at mitigating mould growth, such as frequent cleaning of wheels [2]; however, since fungal spores have an airborne spread, the dispersion of spores in the warehouse can increase, with further contamination of wheels, already micro-damaged on the surface by the cleaning technique [3].

Among the cleaning techniques used before marketing of hard cheeses, dry and wet cleaning represent the most widely used processes and, for this reason, these techniques were considered here. The results demonstrated that, when the techniques were correctly applied on hard cheese wheels, the obtained grated cheeses presented low levels of OTA and STC contamination. Furthermore, the analyses performed on the rind powders removed by dry cleaning demonstrated that the flat side of wheels shows a significantly higher OTA and STC contamination than the round one. This information could be useful when a high contamination of rind wheel is suspected. In conclusion, this work provides new insights into the potential co-occurrence of OTA and STC in grated cheese, food that is widely used in some countries, thus representing a potential risk for consumers’ health.

## 4. Materials and Methods

### 4.1. Reagents and Standards

Chemicals and solvents used for the extraction and clean-up solutions were ACS grade or equivalent (Carlo Erba, Milan, Italy). For HPLC analysis, methanol, acetonitrile, formic and acetic acid were HPLC grade (Merck, Darmstadt, Germany), and water was purified through a Milli-Q treatment system (Millipore, Bedford, MA, USA). Phosphate-buffered saline (PBS) was prepared as follows: NaCl 8 g L^−1^, KCl 0.2 g L^−1^, Na_2_HPO_4_ 1.15 g L^−1^, KH_2_PO_4_ 0.2 g L^−1^, pH 7.4. OTA and STC standards were obtained from Sigma-Aldrich (St. Louis, MO, USA), and the internal standard [^13^C_18_]-sterigmatocystin (96.4% ^13^C) was purchased from Biopure (Tulln, Austria) as standard solution in acetonitrile (1.2 mL, 25.7 µg mL^−1^, uncertainty 1.02 µg mL^−1^). A solution of OTA (40 µg mL^−1^ in benzene-acetic acid 99 + 1 *v*/*v*) was calibrated spectrophotometrically at 333 nm using the value of 5550 L mol^−1^ cm^−1^ for the absorption coefficient (AOAC, 1995) [23]. Working standards were prepared by evaporating an exact volume under a stream of nitrogen and re-dissolving the residue in the HPLC mobile phase. A stock STC standard solution was prepared in ethanol at a concentration of 10 mg L^−1^, and the solution was calibrated spectrophotometrically at 325 nm using the value of 16,218 L mol^−1^ cm^−1^ for the absorption coefficient and stored at −20 °C when not in use. Working standard solutions were prepared by dilution with acetonitrile-water (40/60 *v*/*v*). Five STC standards, mixed with isotopically labelled STC standard solution (12 µg L^−1^; 90 + 10 *v*/*v*), were injected. All solutions were stored at –20 °C when not in use.

### 4.2. Occurrence of OTA and STC in Grated Hard Cheese Samples

For the survey, a total of 107 samples of grated cheese, produced from hard grana-type cheeses, were purchased in several supermarkets in Northern Italy from May 2018 to October 2020. All the samples were kept at −20 °C until the time of analysis. After OTA and STC quantification, results < LOD were considered as ½LOD in calculating mean values.

### 4.3. Mycotoxin Migration from Surface into Inner Parts of the Rind

**Figure 2:**
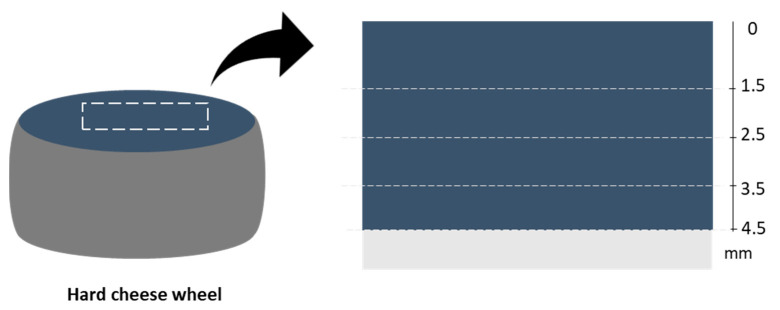
Schematic representation of rind sampling for mycotoxin penetration evaluation.

Rind sampling was carried out in a warehouse where a high mycotoxin contamination on the surface of hard cheeses had previously been detected. Rind samples (n = 16) were taken monthly from wheels of hard grana-type cheese ripened for 13–16 months. Each rind sample had a thickness of 4–5 cm and weighted about 700 g, and samples looked normal, without any visible spoilage. In order to evaluate the migration of mycotoxins from the surface to inner parts of the rind, from each rind sample two pieces of about 12 × 25 cm were portioned and destined to the OTA and STC migration study, and four layers were scraped from the surface to inner parts with a truffle slicer, as shown in Figure 2. 

Firstly, 1.5 mm was removed, and then, further below, three 1.0 mm-thick layers were cut. All the samples were kept at −20 °C until the time of analysis. The analysis on grated samples was then performed in duplicate. 

### 4.4. Cleaning Techniques of Hard Cheese Wheels 

In order to evaluate the efficiency of the main industrial cleaning techniques in reducing mould and mycotoxin contamination of hard cheese surfaces, two different procedures were evaluated, using plants located in a factory for grated cheese production. Each technique was applied on 5 wheels coming from a storehouse in which a remarkable mycotoxin contamination of cheese surface was previously detected. The first method was wet cleaning of wheels with warm water. At the start of the test, the plant had been working for 5 h. It used about 3000 kg of water at 80 °C, which was changed daily, and an aliquot of washing water was sampled before and after washing the 5 wheels and analysed for OTA and STC quantification. After drying, the wheels were horizontally divided and mechanically grated. Samples of grated cheese (about 200 g) were collected every minute for a period of 20 min during the grating process. Five global samples were obtained by mixing the grated cheese samples, in order to represent the five wheels. Before the start of the trial, five samples of grated cheese were collected during normal operation of the plant, in order to compare the data of the trial with those of commercial production. All the samples were analysed for OTA and STC quantification.

The second method was dry cleaning. The wheels were scraped and brushed by an automatic cleaning machine; in this process, about 0.2 mm of rind was removed. The material removed was collected, homogenised and sampled for OTA and STC analysis. Then, the cleaned wheels were grated, and samples were collected as described above for the wet cleaning.

### 4.5. Mycotoxin Analysis

#### 4.5.1. OTA Quantification

OTA was extracted from an aliquot of 10 g of sample with 100 mL of 0.13 M sodium bicarbonate-methanol (50 + 50 *v*/*v*) for 45 min, using a rotary-shaking stirrer, according to the method of Pietri et al. [24]. After filtration through a folded filter paper, an aliquot of the filtrate (5 mL) was diluted with PBS (50 mL) and purified through an immunoaffinity column (Ochratest, WB, Vicam, Watertown, MA, USA). The column was washed with PBS (2 mL) and OTA was slowly eluted (0.5 mL min^−1^) with acetonitrile (3 mL) into a graduated glass vial. The eluate was concentrated under a gentle stream of nitrogen, brought to 2 mL with acetonitrile:water (41 + 59 *v*/*v*) and vortex-mixed for a few seconds. The extract was filtered (Millex HV 0.45 mm, Millipore) before HPLC analysis. The HPLC system consisted of a Perkin Elmer 200 pump (Perkin Elmer, Norwalk, CT, USA), equipped with an AS 1555 sampling system (Jasco Corporation, Tokyo, Japan) and an FP 1520 fluorescence detector (Jasco Corp.) set at 333 nm excitation and 470 nm emission wavelengths. The system was governed by Borwin 1.5 software (Jasco). OTA was separated on a Phenyl-hexyl column (5 µm particle size, 150 × 4.6 mm i.d., Phenomenex, Torrance, CA, USA) at ambient temperature, with a mobile phase gradient acetonitrile−2% acetic acid aqueous solution from 35:65 to 67:33 in 15 min, and the flow rate was 1.0 mL min^−1^. The injection volume was 30 µL.

#### 4.5.2. STC Quantification

STC was extracted from an aliquot of 10 g of sample with 50 mL of acetonitrile:water 80 + 20 *v*/*v*, using a rotary-shaking stirrer for 60 min, according to the method of Marley et al. [25]. After filtration through a folded filter paper, an aliquot of the filtrate (2 mL) was diluted with 20 mL of PBS and cleaned using an immunoaffinity column (R-Biopharm-Rhône, Glasgow, UK). After washing of the column with 2 mL of water, STC was eluted in a graduated glass vial with 6 mL of acetonitrile. The extract was concentrated under a gentle flow of nitrogen and brought to 1 mL with acetonitrile:water 40 + 60 *v*/*v*. An aliquot of 900 µL of cleaned extract was transferred into an autosampler vial and mixed with 100 µL of isotopically labelled STC (12 µg L^−1^). A volume of 20 µL of the extract was injected into an LC-MS/MS system consisting of a LC 1.4 Surveyor pump, a Quantum Discovery Max triple-quadrupole mass spectrometer (Thermo-Fisher Scientific, San Jose, CA, USA) and a PAL 1.3.1 sampling system (CTC Analytics AG, Zwingen, Switzerland). The system was controlled by Xcalibur 1.4 software (Thermo-Fisher). After separation on a Betasil RP−18 column (5 µm particle size, 150 × 2.1 mm, Thermo-Fisher) with a gradient acetonitrile:water (both acidified with 0.2% formic acid; flow rate 0.2 mL min^−1^), the ionisation was performed using positive atmospheric pressure chemical ionisation (APCI), as follows: voltage 4.0 kV, sheath and auxiliary gas 29 and 5 psi, respectively, temperature of the heated capillary 270 °C. The mass spectrometric analysis was performed in selected reaction monitoring (SRM) mode. For fragmentation of the [M + H]^+^ ions (*m*/*z* 325 and 343 for STC and [^13^C_18_]-STC, respectively), argon was used as a collision gas at the pressure of 1.5 mTorr. For STC, three transitions were measured: *m*/*z* 310 (24 V) (quantifier), 281 and 253 (35 V) (qualifiers). For the isotopic label, the transitions were: *m*/*z* 327 (24 V) (quantifier), 297 and 268 (35 V) (qualifiers). 

#### 4.5.3. Performance Evaluation

OTA and STC solutions in solvent, blanks and spiked samples were used to check the performance of the adopted analytical method. Recovery percentages were evaluated by spiking a known blank cheese sample (3 replicates) at 2 levels for each mycotoxin: 2.0 and 10.0 µg kg^−1^ for OTA, and 0.5 and 2.0 µg kg^−1^ for STC. For STC determination, internal standard [^13^C_18_]-STC was added to all sample extracts and calibration standards before the instrumental analysis, in order to compensate the matrix effect which may occur due to the presence of compounds in extract that can co-elute with the analyte, affecting the ionisation of the analyte. The matrix effect can be compensated using the isotopically labelled STC as an internal standard. By normalising the response of STC for its labelled internal standard, it is possible to use solvent standards for calibration of the different products analysed. The limit of detection (LOD) and of quantification (LOQ) were determined by the signal-to-noise approach, defined at those levels resulting in signal-to-noise ratios of 3 and 10, respectively. The analytical response and the chromatographic noise were both measured from the chromatogram of a blank sample extract to which an appropriate volume of standard solutions had been added for each mycotoxin. The linearity of the instrumental analyses was established through five calibration standards in solvent, between 0.075 and 5.0 µg L^−1^ for OTA, and 0.05 and 2.0 µg L^−1^ for STC. The respective calibration curves were generated, and the coefficients of determination were calculated. The linearity of the calibration curves was considered satisfactory if R^2^ > 0.99.

#### 4.5.4. Statistical Analysis

Data are expressed as mean ± standard deviation. Statistical analyses were performed using SPSS v.23 (SPSS Inc., Armonk, NY, USA, 2012). The mycotoxin data regarding the cleaning trials were statistically compared by one-way ANOVA and Tukey’s post hoc test, in order to highlight the significant differences between means. Significant differences were compared at a level of *p* < 0.01.

## Figures and Tables

**Table 1:** Frequency of occurrence, mean ± standard deviation (SD), median, 95th percentile, range of concentration and coefficient of variation of OTA and STC in grated cheese samples collected in Northern Italy.

	Positives	Mean ± SD µg kg^−1^	Median µg kg^−1^	95th Percentile	Range µg kg^−1^	Coefficient of Variation
OTA	52/107	1.80 ± 4.14	<LOD	9.67	<LOD–25.05	2.3
STC	101/107	0.83 ± 1.02	0.47	2.71	<LOD–6.87	1.2

**Table 2:** Concentration (µg kg^−1^) of OTA and STC in layers removed from rind of ripened hard cheese wheels. Na: not analysed.

Layer	Ripened Hard Cheese Wheels															
mm	1	2	3	4	5	6	7	8	9	10	11	12	13	14	15	16
**OTA**																
**0–1.5**	69.9 ± 0.8	291.2 ± 1.3	92.5 ± 0.6	55.8 ± 0.5	370.4 ± 1.6	204.8 ± 2.5	238.1 ± 0.9	85.1 ± 2.1	45.6 ± 0.4	61.1 ± 2.2	34.5 ± 0.7	71.5 ± 1.7	10.8 ± 0.5	55.9 ± 0.9	109.8 ± 3.1	19.5 ± 1.1
**1.5–2.5**	4.82 ± 0.02	87 ± 0.7	10.1 ± 0.3	7.45 ± 0.6	168.5 ± 0.9	72.6 ± 1.2	50.5 ± 1.1	19.2 ± 0.7	5.55 ± 0.3	7.8 ± 1.1	8.25 ± 0.2	15.5 ± 2.1	3.1 ± 0.1	7.7 ± 0.8	32.6 ± 1.4	2.55 ± 0.4
**2.5–3.5**	<0.05	3.2 ± 0.0	1.3 ± 0.1	<0.05	36.5 ± 0.1	6.9 ± 0.9	6.3 ± 0.8	2.45 ± 0.1	1.65 ± 0.8	0.6 ± 0.0	1 ± 0.1	2.45 ± 0.5	0.25 ± 0.0	1.75 ± 0.0	2.1 ± 0.8	0.6 ± 0.0
**3.5–4.5**	Na	<0.05	<0.05	Na	1.15 ± 0.1	1.9 ± 0.6	0.1 ± 0.0	<0.05	<0.05	<0.05	<0.05	<0.05	<0.05	<0.05	0.9 ± 0.2	0.3 ± 0.0
**STC**																
**0–1.5**	2.7 ± 0.1	19 ± 0.7	6.7 ± 0.4	2.6 ± 0.2	15.2 ± 0.7	20.1 ± 0.6	4.5 ± 0.4	14.7 ± 0.4	14.2 ± 0.1	26.3 ± 0.7	14.3 ± 0.2	16.2 ± 0.9	3.1 ± 0.2	20.9 ± 0.7	24.4 ± 0.5	11 ± 0.1
**1.5–2.5**	0.81 ± 0.01	10.1 ± 0.4	2.2 ± 0.1	1.5 ± 0.1	4.1 ± 0.2	10.8 ± 0.4	2.5 ± 0.1	11.8 ± 0.4	10.3 ± 0.4	10.1 ± 0.1	6.2 ± 0.4	8.5 ± 0.4	1.1 ± 0.1	14 ± 0.3	17.8 ± 0.3	5.8 ± 0.5
**2.5–3.5**	<0.05	5.9 ± 0.5	<0.05	<0.05	1 ± 0.1	4.3 ± 0.5	0.5 ± 0.1	<0.05	4.8 ± 0.6	5.4 ± 0.2	4.4 ± 0.2	4.3 ± 0.6	0.9 ± 0.0	6.2 ± 0.3	7.7 ± 0.5	1.4 ± 0.0
**3.5–4.5**	Na	1.2 ± 0.1	Na	Na	0.4	1.2 ± 0.1	0.2 ± 0.0	Na	<0.05	<0.05	3 ± 0.2	2.7 ± 0.1	0.8 ± 0.0	4.4 ± 0.1	5.5 ± 0.2	0.4 ± 0.0

**Table 3:** OTA and STC levels in washing water and in grated hard cheese samples after wet cleaning.

Wet Cleaning Technique	OTA µg kg^−1^	STC µg kg^−1^
Washing water T0	0.44 ± 0.03	<0.05
Washing water T1	0.39 ± 0.07	<0.05
Grated cheese 1	<0.05	0.07 ± 0.00
Grated cheese 2	0.19 ± 0.01	0.10 ± 0.02
Grated cheese 3	0.74 ± 0.11	0.49 ± 0.074
Grated cheese 4	0.42 ± 0.80	0.10 ± 0.03
Grated cheese 5	<0.05	0.10 ± 0.03

T0, T1 = cleaning water sampled before and after wheel cleaning, respectively.

**Table 4:** OTA and STC levels in rind powders removed from the five wheels during the dry-cleaning process.

	OTA µg/kg^−1^			STC µg/kg^−1^		
Dry Cleaning Techniques	Flat Side Scraping	Round Side Scraping	Brushing	Flat Side Scraping	Round Side Scraping	Brushing
Wheel 1	29.4 ± 1.1	1.7 ± 0.8	5.1 ± 0.1	16.3 ± 1.9	0.4 ± 0.0	0.7 ± 0.1
Wheel 2	34.5 ± 0.5	1.2 ± 0.1	1.6 ± 0.3	1.40 ± 0.1	8.8 ± 0.4	0.5 ± 0.1
Wheel 3	112.7 ± 0.9	2.9 ± 0.4	5.2 ± 0.4	2.8 ± 0.8	0.3 ± 0.0	0.4 ± 0.0
Wheel 4	17.9 ± 1.4	2.7 ± 1.0	0.7 ± 0.0	3.5 ± 0.2	0.3 ± 0.1	0.4 ± 0.0
Wheel 5	20.0 ± 0.2	3.1 ± 0.4	6.8 ± 0.7	7.7 ± 0.7	1.1 ± 0.7	1.3 ± 0.7

**Table 5:** OTA and STC levels in the wheels grated after dry cleaning.

Dry Cleaning Techniques	OTA µg kg^−1^	STC µg kg^−1^
Grated cheese 1	0.16 ± 0.02	0.25 ± 0.01
Grated cheese 2	0.45 ± 0.05	0.11 ± 0.01
Grated cheese 3	<0.05	0.05 ± 0.00
Grated cheese 4	<0.05	0.33 ± 0.09
Grated cheese 5	<0.05	0.07 ± 0.00

## Data Availability

The data presented in this study are available in this article.
